# Editorial: An era of personalized medicine in breast cancer: integrating artificial intelligence into practice

**DOI:** 10.3389/fonc.2023.1164808

**Published:** 2023-06-06

**Authors:** Nosheen Masood, San-Gang Wu

**Affiliations:** ^1^ Department of Biotechnology, Fatima Jinnah Women University, Rawalpindi, Pakistan; ^2^ Department of Radiation Oncology, First Affiliated Hospital of Xiamen University, Xiamen, China

**Keywords:** artificial intelligence, personalized medicine, clinical tumor prediction, precision medicine, breast cancer

Breast cancer incidence rate is increasing rapidly despite the advancements made in this field. Hereditary, genetic, and obesity involvement, and the use of alcohol and contraceptives are a few of the main culprits for this disease. However, despite all the enormous research on this disease, cases continue to increase and the rate of recurrence and metastasis remains high. Late diagnosis, wrong diagnosis, and lack of personalized medicine make the condition much worse. For this purpose, there is a need to inculcate artificial intelligence into breast cancer diagnosis and treatment to decrease the rate of mortality and recurrence. The key to breast cancer cure lies in early detection, but there are always problems associated with early diagnosis. One of the major complications associated with a breast cancer diagnosis is to differentiate between carcinoma and other diseases. For this purpose, Zhou et al. developed a deep learning-based artificial intelligence automatic classification system to differentiate breast cancer malignancy from non-lactating mastitis. They found that the interpretations by the senior physician and AI module were consistent with postoperative pathological diagnosis whereas an intermediate aged physician’s interpretations of images were not consistent with postoperative diagnosis, making this method a reliable auxiliary method to distinguish between the two types of diseases with accuracy and high specificity. Breast cancer screening is accompanied by radiological images; therefore, Zhang et al. designed a study to use artificial intelligence for the detection of breast cancer. The CNN (Convolutional Neural Network) algorithm using internal and external validation datasets was used for a total of 2,538 ABUS (automated whole-breast ultrasound) images that showed a good efficiency for BIRAD 4 and 5 compared with BIRAD 2 and 3 lesions. They also concluded that lesions greater than 10 mm were better detected. Desai et al. (2021) and Nomani et al. (2022) also reported that CNN gives higher accuracy for diagnosis and detection of breast cancer. Continuous contrast-enhanced ultrasound allows radiologists to access the dynamic vascularization of vessels and tissues in real time, and this application is exploited for differentiating benign and malignant tissues using machine learning models. The 3D-ResNet-50 model and the XGBoost (Extreme gradient boost) model were best for classifying the tumor for the radiologists using CNN models (Zhu et al.). Lin et al. found that a CEUS-based (continuing education units) nomogram along with the evaluation of clinical features of breast cancer patients cannot differentiate HER2 higher-expressing breast cancers from others. Three models using preoperative CEUS reports that are divided into training and validation groups were analyzed for the study. A machine learning model-based study was conducted by Zhang et al. to reveal the association between ultrasound and early detection of sentinel lymph node status in breast cancer. For this purpose, 10 machine learning algorithms that are already available were used to predict the best diagnostic tool. It was found that the XGBoost model was best for SLN (sentinel lymph node) detection and for preoperative clinical guidance, and SHAP (SHapley Additive exPlanations) was the best tool for a visual picture. Prediction of lymph node metastasis can be done by different machine learning tools. A study on breast invasive micropapillary carcinoma was conducted to find the best model for the prediction of lymph node metastasis. The XGBoost model combined with SHAP was better than LR-based nomogram models. Tumor size was important for detection (Jiang et al.).

In addition to AI, laboratory experiments were conducted in case reports revealing genetic association with breast cancer. One of the case reports by Eskandarion et al. revealed that patients suffering from breast cancer had mutated p4EBP1, PTEN, and TP53 genes leading to the activation of the mTOR signaling pathway. She was given bevacizumab due to profiling and showed reduced liver metastasis followed by mastectomy. Drugs targeting CDK4/6 inhibitor in HR+/HER2− metastasized breast cancer patients need to be evaluated for their effectiveness. Another case study showed that the use of PI3K inhibitors requires more research in advanced breast stages using NGS to arrive at a conclusion (Mao et al.). Huang et al. conducted a meta-analysis to reveal the efficacy of taxane administered to triple-negative breast cancer patients. Taxane monotherapy was compared with taxane-based combination therapies, and it was found that the latter showed more promising results. Taxane-based combination therapies are more effective and well tolerated in advanced-stage triple-negative breast cancer patients. For effective treatment strategy, the size and volume of breast cancer tumors are important; time-consuming and confusing variations in radiologists’ reports exist. The Res-UNet convolutional neural network was used on MRI and the results were promising as they showed that it was effective in clinical decision-making for breast cancer (Yue et al.). Most breast cancer patients undergo chemotherapy followed by radiotherapy. Radiotherapy though seems less invasive but has its consequences. A 3D printed bolus is used in postmastectomy patients undergoing radiotherapy to increase dose effectivity in radiotherapy. It was found that a 3D printed bolus is more precise and caused less acute skin toxicity (Wang et al.). Jeibouei et al. wanted to evaluate the role of seroma analysis after intraoperative radiotherapy in early breast cancer. No conclusion can be deduced from the analysis of seroma studies to decipher the effectiveness of IORT because of the variation in body reactions of patients. It seems that it is related to the immune system and probably unknown or rarely studied factors in this area, such as microbiota in the body of patients. It was suggested that the question can be answered through a trial with the clinical endpoint. Radiation-induced dermatitis of grade greater than or equal to 2 can be predicted in breast cancer patients with high accuracy using multi-region dose-gradient GBDT (gradient-boosted decision trees) with a random-based forest-based encapsulation screening method. It was proved through a study by Feng et al., where they used CT images and clinical and dosimetric details of 214 breast cancer patients.

For the facilitation of breast cancer patients, Liang et al. attempted to create a breast cancer management information platform module based on patient-perceived value. The model was comprehensive and reasonable and proved to be efficient for meeting the healthcare needs of breast cancer. The module is applicable as it provides a platform for the development of subsequent modules. To reach a conclusion, this Research Topic gives detailed information regarding the use of artificial intelligence in practice starting from screening to diagnosis to personalized treatment to management models. In short, this information is interesting, informative, and applicable for oncologists and patients.

## Author contributions

NM has been involved in the write-up of the editorial. S-GW proofread the editorial sent through email. All authors contributed to the article and approved the submitted version.

